# Heterogeneity in PD-L1 expression in malignant peritoneal mesothelioma with systemic or intraperitoneal chemotherapy

**DOI:** 10.1038/s41416-020-01130-x

**Published:** 2020-10-26

**Authors:** Michael G. White, Jefree J. Schulte, Lai Xue, Yaniv Berger, Darryl Schuitevoerder, Charles C. Vining, Hedy L. Kindler, Aliya Husain, Kiran K. Turaga, Oliver S. Eng

**Affiliations:** 1 Department of Surgical Oncology, The University of Texas MD Anderson Cancer Center, Houston, TX USA; 2 Department of Pathology, University of Chicago, Chicago, IL USA; 3 Department of Surgery, Section of General Surgery and Surgical Oncology, University of Chicago, Chicago, IL USA; 4 Department of Medicine, Section of Hematology/Oncology, University of Chicago, Chicago, IL USA

**Keywords:** Mesothelioma, Surgical oncology, Predictive markers

## Abstract

Programmed death-ligand 1 (PD-L1) expression has been described in patients with malignant peritoneal mesothelioma (MPM), but treatment strategies utilising immune checkpoint inhibition are yet to be defined. Here, we examine levels of PD-L1 expression in MPM patients treated with systemic and/or intraperitoneal chemotherapy using tissue from patient tumour biopsies or resections at multiple time points. We found the mean PD-L1 expression was higher in those with a germline mutation and/or those with a higher somatic mutation burden. Moreover, PD-L1 expression was lower in patients who had received prior chemotherapy as compared to the treatment-naive cohort. Twenty patients who received chemotherapy, either systemic and/or peritoneal, between PD-L1 measurements showed marked heterogeneity. Six (30%) patients demonstrated upregulation of PD-L1, while eight (40%) demonstrated downregulation. Heterogeneity in PD-L1 expression in MPM before and after cytotoxic therapies may present an additional consideration when initiating immune checkpoint inhibition in this rare and challenging disease.

## Background

Malignant peritoneal mesothelioma (MPM) is a solid malignancy arising from the mesothelial lining of the peritoneal cavity. Despite aggressive surgical and medical management, patients’ prognosis remains guarded, with a 5-year survival of 42%.^1^ Recently, programmed death-ligand 1 (PD-L1) expression has been noted in a significant proportion of MPM specimens.^2^ These results were translated to a Phase 2 trial of malignant pleural or peritoneal mesothelioma patients treated with checkpoint inhibition with anti-PD-1 therapy. A correlation with higher response rate and durable progression-free survival was demonstrated with increasing PD-L1 expression, although response rates in the peritoneal cohort remained low (12.5%).^3^ The reason behind the low rate of response in the peritoneal cohort remains unclear.

PD-1 inhibition can improve outcomes in various solid malignancies, including mesothelioma. The majority of trials have relied on treating malignancies staining positive for PD-L1, with response rates correlating with the degree of PD-L1 expression.^4,5^ PD-L1 expression, however, has been shown to be variable over time and may be altered by the physiologic pressure of cytotoxic chemotherapy.^6^ The effect of treatment with either systemic chemotherapy or hyperthermic intraperitoneal chemotherapy (HIPEC) on PD-L1 expression in MPM is unclear.

Here, we report MPM patients with quantified levels of PD-L1 expression before and after treatment with systemic chemotherapy and/or HIPEC, characterising baseline levels of expression and correlating PD-L1 levels with demographic and/or pathologic factors. We hypothesise that there is temporal heterogeneity in PD-L1 expression for patients with MPM receiving cytotoxic chemotherapy.

## Methods

This is a retrospective review of an Institutional Review Board-approved (IRB19-0793) single institution database of patients with MPM treated between 2017 and 2019. Patients with available banked tissue underwent immunohistochemical (IHC) staining and were included in the study.

PD-L1 IHC was performed on one representative section with adequate cellularity (defined as a minimum of 100 viable tumour cells present) and minimal necrosis from each case. After deparaffinisation and rehydration, the tissue slides underwent pressure cooker retrieval at pH 9. Dilution (1:50) of anti-PD-L1 antibody (rabbit monoclonal, clone 28.8, Abcam) was applied on the slides utilising Bond III by Leica Biosystems for 25 min and Refine detection by Leica Biosystems. The slides were counterstained and glass coverslipped. Each PD-L1 IHC slide was interpreted by two pathologists simultaneously (J.J.S., A.H.). The percentage of tumour cells with cytoplasmic membranous expression for anti-PD-L1 was reported. Granular cytoplasmic staining was regarded as negative. Somatic mutations were identified using the OncoPlus 1200 Gene panel; a next-generation sequencing assay using the HiSeq 2500 system (Illumina) with variant calling of 147 reportable genes.^7^ Saliva or peripheral blood was sequenced to identify germline variants using a customised targeted gene panel of 85 cancer susceptibility genes as previously described.^8^ Demographic and clinical characteristics associated with PD-L1 status were studied using a Wilcoxon’s rank-sum test, *t* test, or *χ*^2^ test where appropriate.

## Results

In total, 53 patients were studied. Thirty-four (65%) patients expressed PD-L1 (>1% staining), and 9 (17%) had high levels of PD-L1 expression.^5^ After multidisciplinary tumour board review, the majority of patients underwent standard platinum-based systemic chemotherapy along with Pemetrexed. No patients in the cohort received immunotherapy. Clinicodemographic factors associated with PD-L1 expression within 78 specimens from the 53 patients are summarised in Table 1. Higher levels of PD-L1 expression were noted in samples from patients with germline mutations (20% vs. 1%, *p* = 0.04) and higher somatic mutational burdens, defined as >1 mutation per mega base pair (7.5% vs. 0%, *p* = 0.04). Conversely, patients who received systemic chemotherapy prior to biopsy had significantly lower rates of PD-L1 expression (6% vs. 16%, *p* = 0.02). Rates of PD-L1 expression by prior treatment modality showed no difference between the treatment-naive and HIPEC groups (16.1% vs. 13.8%, *p* = 0.86). There was a significant difference between the treatment-naive group and those who had received prior systemic chemotherapy, regardless of HIPEC status (15.8% vs. 5.2%, *p* = 0.04).

**Table 1:** Factors associated with PD-L1 expression in 78 pathological specimens from 53 MPM patients.

Characteristic	No. of specimens	PD-L1%	*P* value
Patient age at diagnosis			0.28
>60 years	36	9.7 (0–88)	
≤60 years	42	14.8 (0–90)	
Patient gender			0.41
Male	51	13.9 (0–90)	
Female	27	9.8 (0–60)	
Histological subtype			0.93
Epithelioid	73	12.3 (0–90)	
Biphasic	5	13.2 (0–60)	
Nuclear grade score			0.32
1	35	14.7 (0–88)	
>1	38	9.9 (0–90)	
Nuclear atypia score			0.63
1	11	14.4 (0–60)	
>1	60	11.7 (0–90)	
Mitotic count score			0.25
1	34	15.1 (0–88)	
>1	38	9.6 (0–90)	
Necrosis			0.55
Yes	18	12.8 (0–60)	
No	55	9.4 (0–90)	
BAP1 protein IHC			0.16
Lost	35	10.8 (0–88)	
Retained	15	19.3 (0–90)	
Germline mutation			**0.04**
Yes	13	20 (0–60)	
No	53	1 (0–90)	
Somatic tumour mutation burden			**0.04**
=1 per Mb	8	0 (0–20)	
>1 per Mb	20	7.5 (0–88)	
Prior chemotherapy (within 2 years)			**0.02**
No chemotherapy	49	16 (0–90)	
Systemic chemotherapy	15	6 (0–50)	
Intraperitoneal chemotherapy	4		
Systemic + intraperitoneal	10		

*Mb* mega base pair.

Bold values indicate statistical significance *p* < 0.05.

Of the 53 MPM patients studied, 20 (38%) had PD-L1 expression measured before and after receiving either systemic or intraperitoneal cytotoxic chemotherapy between biopsies. Of these patients, seven (35%) patients had <1% staining before and after treatment. Six (30%) patients demonstrated PD-L1 upregulation, while eight (40%) demonstrated downregulation after receiving therapy. Two (10)% patients with high (>50%) staining transitioned to low or no staining after HIPEC, while one (5%) patient with high staining transitioned to low staining after systemic chemotherapy.

## Discussion

This is the first study to examine rates of PD-L1 expression within MPM demonstrating significant heterogeneity in expression between paired samples before and after treatment with systemic chemotherapy and/or HIPEC. We showed that in a cohort of MPM specimens, a significant proportion had high levels of staining in at least one biopsy. Rates of staining correlated with germline and somatic mutational burden, but were inversely associated with prior treatment with systemic cytotoxic chemotherapy. Previous work has shown PD-L1 staining to be variable between primary tumour sites and histologies with significant variation within single histologies or even intratumoural variation.^9–11^ Our findings here demonstrate a significant proportion of MPM patients express PD-L1 and may therefore potentially benefit from PD-1 inhibition. Unfortunately, early results of treatment of patients with peritoneal mesothelioma undergoing treatment with PD-1 inhibition demonstrated only a measured response to therapy, significantly less so than in the treatment of pleural mesothelioma.^3^ In the case of pleural mesothelioma, which typically has a more positive response to checkpoint inhibition, current regimens of immunotherapy have failed to show improvement in progression-free or overall survival as compared with conventional therapy in a Phase 3 setting.^12^

Noted alterations in PD-L1 expression after treatment with cytotoxic chemotherapy suggests a potential mechanism for this observed resistance to what would be thought to be a more efficacious therapy. Building on previous preclinical models as well as careful analysis of ongoing trials will ultimately be needed to better define this phenomenon. Prior preclinical work has demonstrated variability with PD-L1 expression under the stress of cytotoxic chemotherapy in mesothelioma cell lines.^13^ These observations raise additional considerations in the context of this measurement of clinically variable PD-L1 expression. First, perhaps the heterogeneity in PD-L1 staining longitudinally within the same patient may explain, in part, the poor response to PD-1 inhibition seen in these patients.^3^ Second, our data suggest the sequence of multimodal treatment of MPM utilising checkpoint inhibition may be of consequence. This is akin to treatment algorithms, which utilise checkpoint inhibition in a neoadjuvant fashion, and may reflect a “window of opportunity” where checkpoint inhibition is optimally effective with cytotoxic chemotherapy or radiation therapy improving responses to immunotherapy.^5,13^ This and our findings should be considered in developing the sequencing of therapies to optimise response rates. Third, it is possible that PD-L1 expression in itself is not a reliable marker for responsiveness to checkpoint inhibition in MPM, and more comprehensive immune panel testing and serum or peritoneal biomarkers may be needed (potentially at multiple time points) to better predict responsiveness to checkpoint inhibition.^11^

Our work is not without significant limitations. As a retrospective study, all of the limitations inherent in this study design persist. Although treated by the same multidisciplinary team, variability in treatment timing, sequence of interventions and the timing and technique of biopsies exists within this cohort. Moreover, the heterogeneity in PD-L1 staining noted may be a result of spatial heterogeneity among different foci of peritoneal disease. Given that disease phylogeny and spatial heterogeneity of peritoneal disease is not well understood, this is an additional consideration we plan to explore in prospective studies. Moving forward spatial heterogeneity will need to be studied with staining of multiple samples of a single specimen when available. While regular staining of multiple areas of a specimen are possible, the potential to explore the circulating biomarkers as indicators of response to immunotherapy has exciting potential to overcome this spatial heterogeneity and further understand the full potential of immunotherapy may have in treating this patient population.^14^

In this study of 78 patients with MPM, over half of them expressed measurable levels of PD-L1. Moreover, a sizeable (12%) proportion had high levels of staining. There was a significant increase in PD-L1 staining in those patients with a higher somatic mutational burden as well as those patients with known deleterious germline mutations. Although PD-L1 staining is present in a significant proportion of MPM specimens, this level can change drastically with the administration of systemic or peritoneal chemotherapy and temporal change. Future studies examining PD-L1 and the role of PD-1 inhibition in MPM are warranted.

## Data Availability

The data that support the findings of this work are available from the corresponding author upon request.
